# Association of Fluoroquinolone Prescribing Rates With Black Box Warnings from the US Food and Drug Administration

**DOI:** 10.1001/jamanetworkopen.2021.36662

**Published:** 2021-12-01

**Authors:** Ashwini Sankar, Kristi M. Swanson, Jiani Zhou, Anupam Bapu Jena, Joseph S. Ross, Nilay D. Shah, Pinar Karaca-Mandic

**Affiliations:** 1 Carlson School of Management, Department of Finance, University of Minnesota, Minneapolis; 2 Department of Economics, Macalester College, St Paul, Minnesota; 3 Robert D. and Patricia E. Kern Center for the Science of Health Care Delivery, Mayo Clinic, Rochester, Minnesota; 4 School of Public Health, University of Minnesota, Minneapolis; 5 Department of Health Care Policy, Harvard Medical School, Harvard University, Boston, Massachusetts; 6 Yale University, Yale School of Medicine, New Haven, Connecticut; 7 Division of Health Care Policy and Research, Mayo Clinic, Rochester, Minnesota

## Abstract

**Importance:**

In 2013 and 2016, the US Food and Drug Administration (FDA) issued warnings and recommended limited use of fluoroquinolones for patients with certain acute conditions. It is not clear how prescribers have responded to these warnings.

**Objective:**

To analyze changes in prescribing of fluoroquinolones after the 2013 and 2016 FDA warnings and to examine the physician characteristics associated with these changes.

**Design, Setting, and Participants:**

This cross-sectional study used Medicare administrative claims data on Medicare fee-for-service beneficiaries and OneKey data on physicians and their organizations from January 1, 2011, to December 31, 2017. The sample was restricted to outpatient visits for sinusitis, bronchitis, and uncomplicated urinary tract infections. An interrupted time series approach was used to analyze the changes in the prescription rate after each FDA warning. Data analysis was performed between January 1, 2011, and December 31, 2017.

**Interventions:**

Two FDA black box warnings released in August 2013 and July 2016.

**Main Outcomes and Measures:**

The main outcome was an indicator for fluoroquinolone prescriptions in 3 periods: before the 2013 warning (baseline period), after the 2013 warning but before the 2016 warning (postwarning period 1), and after the 2016 warning (postwarning period 2).

**Results:**

The sample comprised 1 238 397 unique patients with a total of 2 720 071 outpatient acute care visits. Of this sample, 848 360 were women (68.5%), and the mean (SD) age was 69.7 (12.6) years. The immediate prescribing levels of fluoroquinolones in postwarning period 1 increased by 3.42 percentage points (95% CI, 3.23-3.62; *P* < .001) and declined by −0.77 percentage points (95% CI, −1.00 to −0.54; *P* < .001) in postwarning period 2. The prescribing trend increased by 0.08 percentage points per month (95% CI, 0.08-0.10; *P* < .001) in postwarning period 1 and 0.06 percentage points per month (95% CI, 0.04-0.08; *P* < .001) in postwarning period 2. In postwarning period 1, the prescribing levels for physicians who were affiliated with hospitals with a top 10th percentile case mix index vs those without such affiliation decreased by −1.13 percentage points (95% CI, −1.92 to −0.34; *P* = .005), whereas the levels for primary care physicians declined by −1.34 percentage points (95% CI, −1.78 to −0.88; *P* < .001) compared with non-primary care physicians in postwarning period 2. Physicians at teaching hospitals were the only ones who showed a decline in prescribing trend in postwarning period 1.

**Conclusions and Relevance:**

This cross-sectional study found an overall decline in prescribing of fluoroquinolones after the release of FDA warnings. Understanding the association of physician and organizational characteristics with fluoroquinolone prescribing behavior may ultimately help to identify mechanisms to improve de-adoption.

## Key Points

**Question:**

Are the 2013 and 2016 US Food and Drug Administration (FDA) recommendations to limit the use of fluoroquinolones for certain acute conditions associated with changes in fluoroquinolone prescribing overall and by physician specialty and organizational type?

**Findings:**

In this cross-sectional study of 1 238 397 patients with sinusitis, bronchitis, or uncomplicated urinary tract infection and a total of 2 720 071 outpatient visits, an overall decline in change over time and an immediate change in fluoroquinolone prescribing was observed after the 2016 FDA warning. Certain physicians, such as primary care physicians, were more responsive to FDA warnings than others.

**Meaning:**

Findings of this study suggest that identifying the association of physician and organizational characteristics with fluoroquinolone prescribing practices could help in developing mechanisms for improving de-adoption.

## Teaser

This cross-sectional study assesses the pattern of antibiotics prescribing for sinusitis, bronchitis, and uncomplicated urinary tract infection before and after US Food and Drug Administration warnings.

## Introduction

Fluoroquinolones are a class of broad-spectrum antibiotics that have been commonly prescribed at increasing rates since they became available in the late 1980s. However, concerns have been raised because they present a risk for toxic effects and potential inappropriate use.^1,2,3^

Fluoroquinolones are associated with serious adverse events affecting the musculoskeletal, peripheral nervous, and central nervous systems,^4^ with more recent evidence of aortic dissection or aneurysm.^5^ In August 2013, after receiving numerous reports of adverse events, the US Food and Drug Administration (FDA) issued a warning that highlighted the risk of irreversible peripheral neuropathy (serious nerve damage). In May and July 2016, the FDA revised its fluoroquinolones black box warning to further address these serious safety issues.^6,7^ With these warnings, the FDA recommended limited use of fluoroquinolones for acute sinusitis, acute exacerbation of chronic bronchitis, and uncomplicated urinary tract infection (UTI), contending that the risks of serious adverse effects generally outweigh the benefits for patients with these conditions.

Even as appropriate antibiotic prescribing became a priority,^8^ evidence is mixed on the ability of FDA warnings to reduce prescribing of this class of drugs for these 3 conditions. Some studies showed that prescribing fluoroquinolones for uncomplicated UTIs, acute sinusitis, and acute bronchitis has not substantially changed after the 2016 warning,^9,10^ calling for better methods to disseminate FDA warnings. By contrast, studies that involved 29 southeastern US hospitals^11^ and a national sample of privately insured patients^12^ reported that inpatient prescribing of fluoroquinolones has been declining consistently in trends and in levels across specialties for certain conditions since the 2013 and 2016 warnings.

Discontinuing the prescription of ineffective drugs is also not uniform across all types of prescribers. Some specialties adopt evidence faster than others.^12^ In addition, there may be systematic differences in prescriber behavior on the basis of hospital characteristics, such as teaching status, ownership, and case mix index (CMI).^13,14^

To our knowledge, the present study was the first to build a patient cohort using a 20% random sample of Medicare fee-for-service beneficiaries. We aimed to analyze changes in prescribing of fluoroquinolones after the 2013 and 2016 FDA warnings as well as to examine the physician characteristics associated with these changes. The analysis focused on 3 periods: before the 2013 warning (baseline period), after the 2013 warning but before the 2016 warning (postwarning period 1), and after the 2016 warning (postwarning period 2). We also sought to ascertain whether variations in de-adoption (defined as the discontinuation of a clinical practice after it was previously adopted, especially because of ineffective and harmful practices)^15,16,17,18,19^ existed across organizational affiliations and specialties, identifying areas wherein inappropriate prescribing has persisted, which can ultimately lead to improved patient safety.

## Methods

This cross-sectional study was deemed exempt from review by the University of Minnesota Institutional Review Board, which waived the informed consent requirement because the data used posed a minimal risk to the privacy of individuals and the study could not be conducted without the waiver or alteration or access to and use of such data. We followed the Strengthening the Reporting of Observational Studies in Epidemiology (*STROBE*) reporting guideline.

### Data Source and Study Sample Construction

We used administrative claims data from a 20% random sample of Medicare fee-for-service beneficiaries from January 1, 2011, to December 31, 2017. We also obtained data on physicians and their organizations from OneKey (IQVIA), a clinician reference database of combined data from IMS Health, SK&A Information Services, and Healthcare Data Solutions.

We constructed a patient-by-month sample in the following manner. First, we identified outpatient evaluation and management visits with a diagnosis of sinusitis, bronchitis, uncomplicated UTI, and other acute respiratory tract infections using the *International Classification of Diseases, Ninth Revision, Clinical Modification* and *International Statistical Classification of Diseases, Tenth Revision, Clinical Modification *diagnosis codes. We required patients to be continuously enrolled in both Parts A and B of Medicare. We further restricted this sample to (1) those with no health maintenance organization or managed care enrollment for the 12 months before the visit, during the month of the visit, and the month after the visit and (2) those with Medicare Part D coverage during the month of the index visit and the next month. To ensure that the identified visit was the index visit for the acute infectious condition of interest, we excluded visits with an outpatient encounter that met the restriction criteria within the 2 weeks before the index visit. We excluded patients with a complicated UTI because it was not 1 of the conditions outlined in the FDA warnings (Table 1). Second, we included indicators for each physician characteristic (ie, primary care physician [PCP]; affiliation with an integrated delivery network [IDN], a for-profit hospital, a teaching hospital, or a hospital with a top 10th percentile CMI).

**Table 1.** Approved Indications for Fluoroquinolones

Diagnosis	*ICD-9* code^a^	*ICD-10* code^a^
Medical history of type 1 or type 2 diabetes	250*	E10*, E11*, Z79.4
Lupus	710*	M32.1*, M32.8, M32.9, L93*
Cancers	140*-239*	C00-D49
HIV	042*, 043*, 044*	B20, Z21
Kidney transplant	V42.0, 55.6, 55.69	Z94.0, 0TY00Z0, 0TY00Z1, 0TY00Z2, 0TY10Z0, 0TY10Z1, 0TY10Z2
Urinary tract obstruction or functional or anatomic abnormality of the urinary tract within 6 mo before diagnosis	599.60, 599.6, 599.69, 753.2*, 753.4, 753.6, 753.8	N13.9, Q62*, Q64.2, Q64.3*, Q64.6, Q64.7*
Indwelling urethral catheter within 6 mo before diagnosis	57.94	Z96.0
Urethral stent within 6 mo before diagnosis	V53.6	Z96.0 (also used for urethral stent), Z46.6
Nephrostomy tube or urinary diversion within 6 mo before diagnosis	V44.6	Z43.6, Z93.6
Chancroid	099.0	A57
Chlamydia	077.98, 078.88, 079.98, 099.41, 099.50-099.59	A74*, N34.1, A56*
Genital herpes	054.10-054.19	A60*
HPV	079.4, 078.10, 078.11, 078.19	B97.7, B07.9, A63.0, B07.8
Molluscum contagiosum	078.0	B08.1
Gonorrhea (excluding PID)	098.0-098.89 (excluding 098.10, 098.17, 098.30, 098.36, 098.37, and 098.39), 647.10-647.14	A54* (excluding A54.29, and A54.24), O98.2*
Granuloma inguinale	099.2	A58
Lymphogranuloma venereum	099.1	A55
PID	614.0-614.2, 098.17, 098.37, 614.3, 614.4, 614.5, 614.7, 098.10, 614.8, 614.9, 098.30, 098.39, 615.0-615.9, 098.36	N70*, A54.29, N73*, N71*
Adult syphilis, all stages	091.0-091.9, 092.0-092.9	A51*
Syphilis	094.0-094.9, 095.0-095.9, 096, 097.0-097.9, 647.0-647.04, 090.0-090.9	A52*, O98.1*, A50*, A53*
Trichomoniasis	131.0-131.9	A59*
Other NGU	099.40, 099.49	N34.1
Venereal diseases	647.2-647.24, 099.8, 099.9	O98.3*, A63.8, A64

Abbreviations: HPV, human papillomavirus; *ICD-9*, *International Classification of Diseases, Ninth Revision*; *ICD-10*, *International Statistical Classification of Diseases and Related Health Problems, Tenth Revision*; NGU, nongonococcal urethritis; PID, pelvic inflammatory disease.

^a^ A wildcard search allowed for an asterisk (*) to be entered to select multiple, valid *ICD-9* or *ICD-10* diagnosis codes when the partial *ICD-9* or *ICD-10* code was entered.

Because this analysis was not a longitudinal study of a cohort of patients, we allowed the patients to reenter the sample if they met the inclusion and exclusion criteria at a later time.

### Primary Outcome

The primary outcome was a dichotomous indicator for fluoroquinolone prescriptions that were filled within 7 days after the index visit, specifically for levofloxacin (Levaquin), ciprofloxacin (Cipro), moxifloxacin hydrochloride (Avelox), ofloxacin (Ocuflox; Floxin), gemifloxacin mesylate (Factive), and delafloxacin meglumine (Baxdela). We identified these antibiotic prescriptions from the Medicare Part D Event file using National Drug Codes and drug names. The changes in prescribing associated with these FDA warnings were expressed as percentage points, which were absolute changes in percentages.

### Physician and Patient Characteristics

We identified the rendering physicians who were associated with eligible index visits using the National Provider Identifier number. We linked the physician and organizational information from the OneKey database to the Medicare claims data using the physicians’ National Provider Identifier numbers.

Using annual OneKey data, we included in the regression model an indicator for PCPs according to their specialty: family medicine, internal medicine, general practice, and geriatric medicine. We also constructed 4 indicator variables for organizational characteristics according to physician affiliations with at least 1 IDN, at least 1 teaching hospital, at least 1 for-profit hospital, or at least 1 hospital with a top 10th percentile CMI for treating patients with conditions that have higher levels of complexity.

Using the Medicare Master Beneficiary Summary File, we set patient age, sex, race and ethnicity (which were based on the administrative enrollment data of Medicare beneficiaries from the Centers for Medicare and Medicaid Services), indicators for all months of the study (to control for any seasonality), and the 4 US Census regions (Northeast, Midwest, South, and West) as the control variables. We also included the Elixhauser Comorbidity Index (the weighted score) to control for any severity of illnesses that could potentially alter prescribing.^20^

### Statistical Analysis

We used an interrupted time series approach to assess the association of the 2013 and 2016 FDA warnings with prescribing of fluoroquinolones. This approach allows for estimating an association immediately as the prescribing level changes and over time as the trend changes^21,22^ after each FDA warning. A technical representation of the empirical specifications is presented in the eAppendix in the Supplement.

We conducted a linear probability model instead of a nonlinear regression model for ease of interpretation.^23^ The 31 months before the 2013 FDA warning was the baseline period. We included a variable with a value of 1 for all of the months after the 2013 warning but before the 2016 warning (postwarning period 1), and we added another indicator variable for the months after the 2016 FDA warning (postwarning period 2). To account for potentially confounding national systematic trends, we incorporated a monthly linear time trend in the analysis. We also used the interactions between each postwarning period indicator variable and the monthly linear time trend. These variables (the indicators for the 2 FDA warnings) and the trend variables interacted with the warnings and composed the main explanatory variables of interest.

We excluded data from 3 months before, 3 months after, and the month of the FDA warning announcement (eTable 6 in the Supplement for the sensitivity analysis with different washout periods). All regression models were adjusted for physician and patient characteristics.

We also examined the association between the prescription of fluoroquinolones after each warning and the status of physicians as a PCP or non-PCP as well as the type of organization in which they practiced. We included variables on physician specialty (PCP or not) and physician affiliation with teaching hospitals, for-profit hospitals, IDNs, and hospitals with a top 10th percentile CMI. We also analyzed changes in trends and levels stratified by each condition (sinusitis, bronchitis, and uncomplicated UTI). We applied clustering of SEs at the patient level to control for any serial correlation of errors. We used the Durbin-Watson test for autocorrelation in the adjusted regression model and found no autocorrelation.

A 2-tailed *P* values from the results were compared with a 95% CI value of 0.05. If *P* < .05, the coefficient was considered to be statistically significant. All statistical calculations and plots were performed with Stata, version 16.1 (StataCorp LLC). Data analysis was performed between January 1, 2011, and December 31, 2017.

## Results

The study cohort consisted of 1 238 397 unique patients with a primary diagnosis of sinusitis, bronchitis, or uncomplicated UTI who underwent an outpatient acute care evaluation and management visit, for a total of 2 720 071 visits. The cohort comprised 848 360 female (68.5%) and 390 037 male (31.5%) patients, with a mean (SD) age of 69.7 (12.6) years. Of these patients, 283 904 (22.9%) lived in the Midwest, 229 430 (18.5%) in the Northeast, 190 510 (15.4%) in the West, and 534 553 (43.2%) in the South. Overall, 86 386 patients (7.0%) were identified under African American, 59 812 (4.8%) under Hispanic, 1 040 771 (84.0%) under non-Hispanic White, and 51 428 (4.2%) under Other (Asian/Pacific Islander, American Indian/Alaska Native, or others) race and ethnic groups (Table 2).

**Table 2.** Characteristics of the Patients and Physicians in the Sample^a^

Variable	No. (%)
No. of visits per patient-month	2 720 071
Total No. of unique patients	1 238 397
Condition	
Sinusitis	728 375
Bronchitis	388 628
Uncomplicated UTI	388 324
Main outcome, mean (SD), %	
Fluoroquinolone prescription rate	8.7 (28.2)
Baseline period prescription rate	0.2 (30.3)
Postwarning period 1 prescription rate	7.4 (26.2)
Postwarning period 2 prescription rate	5.4 (22.7)
Patient characteristics	
Age, mean (SD), y	69.7 (12.6)
Female sex	848 360 (68.5)
Male sex	390 037 (31.5)
Race and ethnicity^b^	
African American	86 386 (7.0)
Hispanic	59 812 (4.8)
White	1 040 771 (84.0)
Other^c^	51 428 (4.2)
Region	
Northeast	229 430 (18.5)
Midwest	283 904 (22.9)
South	534 553 (43.2)
West	190 510 (15.4)
Total No. of unique physicians	170 938
Physician characteristics	
PCP	114 175 (66.8)
General practitioner	5621 (4.9)
Internal medicine specialty	50 824 (44.5)
Family medicine specialty	59 730 (52.3)
Geriatric medicine specialty	1616 (1.4)
Physician affiliation with at least 1 institution	
IDN	162 104 (94.8)
Teaching hospital	112 351 (65.7)
For-profit hospital	66 535 (38.9)
Hospital with top 10th percentile CMI	36 898 (21.6)

Abbreviations: CMI, case mix index; IDN, integrated delivery network; PCP, primary care physician; UTI, urinary tract infection.

^a^ Medicare fee-for-service (20% random sample) and OneKey data were from January 1, 2011, to December 31, 2017.

^b^ Race and ethnicity were based on the administrative enrollment data of Medicare beneficiaries from the Centers for Medicare and Medicaid Services.

^c^ Other included Asian/Pacific Islander, American Indian/Alaska Native, or others.

A total of 170 938 unique physicians prescribed fluoroquinolones during the study period. Of these physicians, 162 104 (94.8%) were affiliated with at least 1 IDN, 112 351 (65.7%) with at least 1 teaching hospital, 66 535 (38.9%) with at least 1 for-profit hospital, and 36 898 (21.6%) with at least 1 hospital with a top 10th percentile CMI. Most physicians were PCPs (114 175 [66.8%]) (Table 2).

### Association of the FDA Warnings With De-adoption of Fluoroquinolones

Table 3 shows the association between the 2013 and 2016 FDA warnings and fluoroquinolone prescriptions, adjusted for physician and patient characteristics. We found a declining trend of prescribing by −0.18 percentage points per month (95% CI, −0.19 to −0.18; *P* < .001) even before the 2013 warning or the baseline period.

**Table 3.** Association of the 2013 and 2016 US Food and Drug Administration Warnings With Fluoroquinolone Prescribing for Indicated Conditions^a^

Observation (n = 2 335 148)^b^	Patients with indicated conditions, coefficient (95% CI), percentage point per month	*P* value
**Observed time trend**		
Baseline trend	−0.18 (−0.19 to −0.18)	<.001
Change in trend		
Postwarning period 1 vs baseline period	0.08 (0.08 to 0.10)	<.001
Postwarning period 2 vs postwarning period 1	0.06 (0.04 to 0.08)	<.001
**Observed change in use levels**		
Change in level		
Postwarning period 1 vs baseline period	3.42 (3.23 to 3.62)	<.001
Postwarning period 2 vs postwarning period 1	−0.77 (−1.00 to −0.54)	<.001

^a^ The dependent variable was a fluoroquinolone prescription indicator; clustered or robust SEs were applied in all models. All regressions controlled for patient age, sex, race and ethnicity, Elixhauser Comorbidity Index score, US Census regions (ie, Northeast, Midwest, South, and West), indicators for months of the study, indicators for primary care physician, and indicators for physician affiliation (ie, integrated delivery network, for-profit hospital, teaching hospital, or hospital with top 10th percentile case mix index). Indicated conditions were acute sinusitis, acute exacerbation of chronic bronchitis, and uncomplicated urinary tract infection (UTI). Patients with complicated UTI conditions were excluded.

^b^ Observed time trend displays the adjusted monthly change in fluoroquinolone use among the Medicare patient sample. Baseline trend is the adjusted monthly time trend in fluoroquinolone prescribing before the 2013 warning; the postwarning period trends are the adjusted monthly time trends after the 2013 and 2016 warnings; change in trend is the difference in these 2 monthly rates of change; and change in level is the difference in monthly use levels, where fluoroquinolone prescribing was adjusted for regional fixed effects, linear time trends, and patient and physician characteristics.

In postwarning period 1, the trend in fluoroquinolone prescribing increased significantly to 0.08 percentage points per month (95% CI, 0.08-0.10; *P* < .001) compared with the baseline period. In postwarning period 2, the trend in fluoroquinolone prescribing further increased from postwarning period 1 by 0.06 percentage points per month (95% CI, 0.04-0.08; *P* < .001). In the analyses that were stratified by condition, the trends for prescribing for patients with sinusitis and bronchitis were the same in postwarning periods 1 and 2. Among patients with uncomplicated UTI, we found that the baseline trend was flat. In postwarning period 1, the trend was −0.04 percentage points per month (95% CI, −0.06 to −0.02; *P* < .001), but the trend in postwarning period 2 was not significant (Figure; eTable 1 in the Supplement). The level of fluoroquinolone prescribing increased in postwarning period 1 by 3.42 percentage points (95% CI, 3.23-3.62; *P* < .001). However, in postwarning period 2, the level of fluoroquinolone prescribing decreased significantly from postwarning period 1 by −0.77 percentage points (95% CI, −1.00 to −0.54; *P* < .001).

**Figure.**
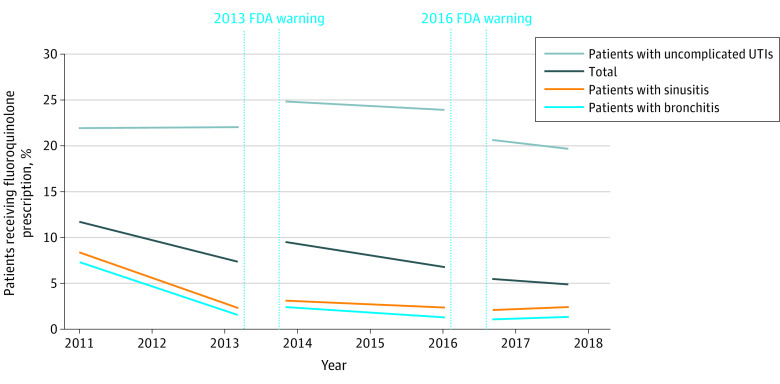
Fluoroquinolone Prescribing Trends Before and After US Food and Drug Administration (FDA) Warnings Each line represents the trend lines from the regression that was adjusted for monthly and regional fixed effects as well as patient and physician characteristics. The total line is the regression with all patients, whereas the other lines are for the samples for each of the 3 conditions (bronchitis, sinusitis, and uncomplicated urinary tract infection [UTI]).

### De-adoption of Fluoroquinolones by Physician Characteristics

Table 4 shows the results of level and trend changes by physician characteristics after the FDA warnings were issued.

**Table 4.** Differential Association of Physician Characteristics With Fluoroquinolone Prescribing for Indicated Conditions^a^

Regression model^b^	Patients with indicated conditions, coefficient (95% CI), percentage point per month	*P* value
**Model 1: Differences for PCPs vs non-PCPs**		
Baseline trend	−0.07 (−0.08 to −0.06)	<.001
Change in trend		
Postwarning period 1 vs baseline period	0.11 (0.08 to 0.12)	<.001
Postwarning period 2 vs postwarning period 1	0.06 (0.02 to 0.09)	.003
Change in level		
Postwarning period 1 vs baseline period	0.69 (0.27 to 1.12)	.001
Postwarning period 2 vs postwarning period 1	−1.34 (−1.78 to −0.88)	<.001
**Model 2: Differences for physicians with vs physicians without IDN affiliation **		
Baseline trend	0.01 (−0.01 to 0.03)	.28
Change in trend		
Postwarning period 1 vs baseline period	−0.00 (−0.04 to 0.03)	.82
Postwarning period 2 vs postwarning period 1	−0.04 (−0.12 to 0.05)	.39
Change in level		
Postwarning period 1 vs baseline period	0.28 (−0.32 to 0.88)	.37
Postwarning period 2 vs postwarning period 1	0.16 (−0.89 to 1.21)	.76
**Model 3: Differences for physicians with vs physicians without teaching hospital affiliation **		
Baseline trend	0.04 (0.04 to 0.06)	<.001
Change in trend		
Postwarning period 1 vs baseline period	−0.06 (−0.08 to −0.04)	<.001
Postwarning period 2 vs postwarning period 1	0.01 (−0.03 to 0.04)	.69
Change in level		
Postwarning period 1 vs baseline period	−0.53 (−0.92 to −0.14)	.008
Postwarning period 2 vs postwarning period 1	0.28 (−0.16 to 0.72)	.21
**Model 4: Differences for physicians with vs physicians without for-profit hospital affiliation **		
Baseline trend	−0.02 (−0.03 to −0.01)	.007
Change in trend		
Postwarning period 1 vs baseline period	0.01 (−0.01 to 0.02)	.39
Postwarning period 2 vs postwarning period 1	0.04 (−0.00 to 0.07)	.07
Change in level		
Postwarning period 1 vs baseline period	0.28 (−0.12 to 0.68)	.17
Postwarning period 2 vs postwarning period 1	−0.06 (−0.52 to 0.38)	.78
**Model 5: Differences for physicians with vs physicians without affiliation with hospital at top 10th percentile CMI **		
Baseline trend	0.04 (0.02 to 0.07)	.003
Change in trend		
Postwarning period 1 vs baseline period	−0.03 (−0.06 to 0.01)	.15
Postwarning period 2 vs postwarning period 1	−0.04 (−0.09 to 0.00)	.06
Change in level		
Postwarning period 1 vs baseline period	−1.13 (−1.92 to −0.34)	.005
Postwarning period 2 vs postwarning period 1	0.26 (−0.34 to 0.86)	.39

Abbreviations: CMI, case mix index; IDN, integrated delivery network; PCP, primary care physician.

^a^ The dependent variable was a fluoroquinolone prescription indicator; clustered or robust SEs were applied in all models. All regressions controlled for patient age, sex, race and ethnicity, Elixhauser score, US Census regions (ie, Northeast, Midwest, South, and West), indicators for months of the study, indicators for primary care physician, and indicators for physician affiliation (ie, IDN, for-profit hospital, teaching hospital, or hospital with top 10th percentile CMI). Indicated conditions were acute sinusitis, acute exacerbation of chronic bronchitis, and uncomplicated urinary tract infection (UTI).

^b^ The regression models shown excluded patients with complicated UTI. Each model included additional interaction terms between the monthly time trends and the indicated physician characteristic. For example, model 1 shows that PCPs had a significantly different rate of fluoroquinolone adoption during the baseline period and had a faster trend in prescription rate in postwarning periods compared with non-PCPs. By contrast, model 3 shows that physicians affiliated with teaching hospitals had a significantly higher trend in adoption rates during the baseline period but had a significantly slower trend in postwarning period prescription rates compared with other physician types. All regressions controlled for patient age; race and ethnicity; physician specialty; physician affiliation with teaching hospital, IDN, for-profit hospital, or hospital with top 10th percentile CMI; and quarterly and regional fixed effects.

In model 1, the fluoroquinolone prescribing trend by month among PCPs compared with other physician specialties was lower by −0.07 percentage points (95% CI, −0.08 to −0.06; *P* < .001) in the baseline period. This trend was reversed after postwarning period 1, increasing by 0.11 percentage points per month (95% CI, 0.08-0.12; *P* < .001) compared with non-PCP prescribing trends. During postwarning period 2, the PCP prescribing trend was 0.06 percentage points per month (95% CI, 0.02-0.09; *P* = .003), which was higher than the non-PCP prescribing trends.

The declining baseline trend for PCPs vs non-PCPs was associated with the decreasing trend in prescribing for sinusitis and bronchitis conditions (eTables 2 to 4 in the Supplement show differential prescribing patterns for each indication). By contrast, patients with an uncomplicated UTI experienced an increase in prescribing trend before the 2013 warning, which continued to increase after the 2013 warning but decreased after the 2016 warning (0.04 [95% CI, 0.00-0.08; *P* = .03] vs −0.17 [95% CI, −0.25 to −0.08; *P* < .001]). The magnitude of the downward prescribing trend in the baseline period for physicians with a for-profit hospital affiliation vs other physicians was lower by −0.02 percentage points per month (95% CI, −0.03 to −0.01; *P* = .007). The differential trends by hospital ownership type, however, disappeared in the postwarning periods.

Physicians who were affiliated with teaching hospitals had a downward prescribing trend in the baseline period, but the magnitude of this trend was higher (ie, the declining trend slowed down, which was unfavorable) by 0.04 percentage points per month (95% CI, 0.04-0.06; *P* < .001) than for those without teaching hospital affiliation. In postwarning period 1, we found a relative decline in trend of −0.06 percentage points per month (95% CI, −0.08 to −0.04; *P* < .001) for physicians who were affiliated with teaching hospitals vs other physicians. However, no significant change in trend in postwarning period 2 was observed compared with postwarning period 1.

The magnitude of the baseline downward trend for physicians who were affiliated with hospitals with a top 10th percentile CMI vs other physicians was higher (unfavorable) by 0.04 percentage points per month (95% CI, 0.02-0.07; *P* = .003). The differential trends, however, were not significant in postwarning period 1 or 2. The IDN-affiliated physicians had no differential trends compared with other physicians.

In analyzing the immediate changes in the levels of prescribing in postwarning periods 1 and 2, we observed that only physicians who were affiliated with teaching hospitals and hospitals with a top 10th percentile CMI had significant decreases in levels compared with other physicians in postwarning period 1. For example, physicians in hospitals with a top 10th percentile CMI had decreased prescribing by −1.13 percentage points (95% CI, −1.92 to −0.34; *P* = .005). With regard to the change in postwarning period 2 from postwarning period 1, PCPs were the only physicians who had a significant decline in prescribing because of significant decreases in prescribing levels for bronchitis and uncomplicated UTI. For example, PCPs showed a −1.34 percentage points (95% CI, −1.78 to −0.88; *P* < .001) decrease.

Furthermore, we analyzed if prescribing by PCPs vs non-PCPs varied by their organizational affiliation (teaching hospital, IDN, for-profit hospital, and hospital with a top 10th percentile case mix index) (eTable 5 in the Supplement). We found from this analysis that the overall trends and levels were consistent for PCPs vs non-PCPs, regardless of their for-profit hospital or IDN setting. For PCPs in teaching hospitals, however, the results showed a higher prescribing trend in the baseline period compared with PCPs in nonteaching hospitals and a lower prescribing trend in postwarning period 1.

## Discussion

In this nationally representative sample of the Medicare fee-for-service population, we found a significant decline in fluoroquinolone prescribing before the 2013 FDA warning, and this decline slowed after both the 2013 and 2016 warnings. The first boxed warning in 2008 for fluoroquinolones might have added caution to physicians’ use of this therapy, which is suggested by the general decline in fluoroquinolone prescribing in the baseline period.^11,24^

Despite repeated warnings, some physicians were not responsive to the recommendations. Although PCPs showed a general decline in prescribing trends compared with non-PCPs, they did not demonstrate a differential change in behavior after the warnings, perhaps because many PCPs wrongly believed that fluoroquinolones were more appropriate for dealing with uncomplicated UTI symptoms than the recommended first-line therapies for this condition.^25^ Physicians who were affiliated with teaching hospitals showed the opposite trend. Their prescribing trend was higher than that for other physicians in the baseline period but decreased significantly during postwarning period 1. This finding is similar to the de-adoption trends of drotrecogin alfa by teaching hospitals, which showed the highest increase in adoption before the release of a major guideline and also the sharpest decline in trend after the guideline was issued.^26^

Fluoroquinolones are not the recommended first-line therapy for sinusitis and uncomplicated UTIs, yet they are among the top 4 most commonly prescribed antibiotic classes.^8,27^ In light of increasing antibiotic resistance,^28,29^ stewardship efforts have targeted inappropriate fluoroquinolone prescribing in adults,^11^ aiming to reduce the routine use of this drug class.^10,30^ Such efforts, in conjunction with the FDA warnings, might explain some of the decline in prescribing behavior over the study period as well as the specific changes in prescribing rates associated with certain physician characteristics.

Some findings from this study, such as the general decline in prescribing trends, are consistent with the results of recent research,^11,24^ and others are novel contributions to the literature. Overall, the results have important policy implications. De-adoption of fluoroquinolones, a potentially unsafe treatment, may help generate strategies to improve patient safety. Furthermore, identifying the association of certain physician and organizational characteristics with fluoroquinolone prescribing may inform the development of mechanisms that make de-adoption faster and more effective.

### Limitations

This study has some limitations. The FDA warnings beginning in 2008 as well as other antibiotic stewardship efforts might have played a role in the fluoroquinolone prescribing trend. Separating the implications of the 2013 and 2016 warnings from those of the earlier warnings and stewardship efforts might not have been possible with the present analysis. Another factor in the decline in fluoroquinolone prescribing was that not all prescribed drugs had a generic version during the study period, which could have changed the affordability for the Medicare beneficiaries in this sample. Although both ciprofloxacin and ofloxacin had branded and generic versions during the entire study period, the other drugs had only generic versions for part of this time. Furthermore, the prescription record in the administrative claims data does not necessarily mean that the medication was actually used. Possible missing data on out-of-pocket medication purchases could also affect the results of the analysis.

Because the FDA warnings were issued at the same time across the US, leaving no control areas that did not receive these warnings, the estimated coefficients from this analysis did not measure the causal impact. In addition, antibiotic sensitivity tends to guide treatment choice, which was not considered in this analysis, even though some of the correlation in treatment choice may have been addressed by the clustered SEs at the patient level. These limitations are nevertheless addressed by the large nationally representative cohort of Medicare beneficiaries that we constructed.

## Conclusions

This cross-sectional study found that the 2013 FDA warning about fluoroquinolone use was associated with a significant decline in fluoroquinolone prescribing trends among physicians who were affiliated with hospitals with a top 10th percentile CMI and teaching hospitals. The 2016 FDA warning, however, was associated with bigger decreases in fluoroquinolone prescribing overall and among PCPs. Identifying the association between physician and organizational characteristics and fluoroquinolone prescribing behavior may inform mechanisms for improving de-adoption.

## Notes

Supplement. **eAppendix.** Statistical Analysis **eTable 1.** Association of FDA Warnings With Fluoroquinolone Prescriptions Across Three Acute Indications **eTable 2.** Differential Association of Physician Affiliations After FDA Warnings With Fluoroquinolone Prescriptions for Patients With Sinusitis **eTable 3.** Differential Association of Physician Affiliations After FDA Warnings With Fluoroquinolone Prescriptions for Patients With Bronchitis **eTable 4.** Differential Association of Physician Affiliations After FDA Warnings With Fluoroquinolone Prescriptions for Patients With Uncomplicated UTI **eTable 5.** Differential Association of PCPs Across Different Institutional Affiliations **eTable 6.** Sensitivity Analysis With Different Washout Periods
