# Supplementary material for: Association of Fluoroquinolone Prescribing Rates With Black Box Warnings from the US Food and Drug Administration
Source: JAMA Netw Open. 2021 Dec 1;4(12):e2136662. doi: 10.1001/jamanetworkopen.2021.36662 (PMC8637256; doi:10.1001/jamanetworkopen.2021.36662)
Supplement: Supplement. — eAppendix. Statistical Analysis eTable 1. Association of FDA Warnings With Fluoroquinolone Prescriptions Across Three Acute Indications eTable 2. Differential Association of Physician Affiliations After FDA Warnings With Fluoroquinolone Prescriptions for Patients With Sinusitis eTable 3. Differential Association of Physician Affiliations After FDA Warnings With Fluoroquinolone Prescriptions for Patients With Bronchitis eTable 4. Differential Association of Physician Affiliations After FDA Warnings With Fluoroquinolone Prescriptions for Patients With Uncomplicated UTI eTable 5. Differential Association of PCPs Across Different Institutional Affiliations eTable 6. Sensitivity Analysis With Different Washout Periods [file jamanetwopen-e2136662-s001.pdf]

Supplementary Online Content

Sankar A, Swanson KM, Zhou J, et al. Association of fluoroquinolone prescribing rates with black box warnings from the US Food and Drug Administration. *JAMA Netw Open*. 2021;4(12):e2136662. doi:10.1001/jamanetworkopen.2021.36662

eAppendix. Statistical Analysis

eTable 1. Association of FDA Warnings With Fluoroquinolone Prescriptions Across Three Acute Indications

eTable 2. Differential Association of Physician Affiliations After FDA Warnings With Fluoroquinolone Prescriptions for Patients With Sinusitis

eTable 3. Differential Association of Physician Affiliations After FDA Warnings With Fluoroquinolone Prescriptions for Patients With Bronchitis

eTable 4. Differential Association of Physician Affiliations After FDA Warnings With Fluoroquinolone Prescriptions for Patients With Uncomplicated UTI

eTable 5. Differential Association of PCPs Across Different Institutional Affiliations

eTable 6. Sensitivity Analysis With Different Washout Periods

This supplementary material has been provided by the authors to give readers additional information about their work.

© 2021 Sankar A et al. *JAMA Network Open.*

eAppendix. Statistical Analysis

We examined the changes in the level and trend of antibiotic prescription after the publication of FDA warnings using the following linear probability model:

$$Y_{ijm} = \sum_{p=1,2} [\, \alpha^p P_m^p + \beta^p (P_m^p \times m)] + \gamma m + \theta Z_{ijm} + \epsilon_{ijm}, \qquad (1)$$

where Y_{ijm} is a dichotomous variable indicating whether physician j prescribed an antibiotic for patient i in month-year m, P_m^p are binary variables that indicate whether month-year m is in period p (i.e., after FDA revision in 2013 and period 2 after the FDA warning in 2016). We excluded data from the month in which the FDA warnings were issued and three months before and afterward as washout periods, in order to avoid potential anticipatory effects. We analyzed other washout periods similar to sensitivity analysis conducted by another study[12] and found that the results were qualitatively and quantitatively similar (see eTable 6 in the SM). We ultimately chose the 7-month washout based on other studies that used the same washout period.[15,16] The vector Z_{ijm} denotes patient and physician characteristics as controls, and ϵ_{ijm} is the error term.

In the first part of the analysis, as represented by Equation (1), the main parameters of interest are γ, β^1, β^2, α^1 and α^2. The coefficient γ represents the general trend in prescribing even before the warning in 2013 happened. In other words, it represents the trend in the baseline period. The coefficients β^p represent the changes in trends in period p in relation to the baseline period as a result of the warning that happened in that month-year. The term $\beta^1 + \beta^2$ represents the difference between the trend after 2016 as compared to the baseline. The coefficients, α^1 and α^2, represent the difference in prescription levels in periods 1 and 2 as compared to that of the baseline.

In the second part of the analysis, as described in Equation (2) below, we interacted both the level and the trend changes with physician characteristics to assess whether there were differences in antibiotic prescription by physician groups and specialty.

$$Y_{ijm} = \sum_{p=1,2} [\, \alpha^p P_m^p + \beta^p (P_m^p \times m) + \delta^p (P_m^p \times m \times C_{ij})] + \eta(m \times C_{ij}) + \gamma m + \sigma C_{ij} + \theta X_{ijm} + \epsilon_{ijm}, \qquad (2)$$

where C_{ij} represents any physician characteristics of interest for whom we would estimate the change in trend after the FDA warning came about, and X_{ij} denotes patient or physician characteristics other than C_{ij}.

Equation (2) allowed us to examine whether there were differential time trends by physician characteristic of interest. The parameters of interest were the time trends and levels. When $C_{ij} = 0$, the changes in levels relative to the baseline period are α^p, and the changes in trends relative to the baseline period are β^p. When $C_{ij} = 1$, the changes in levels and trends relative to the baseline period are $(\sigma + \alpha^p)$ and $(\eta + \beta^p)$, respectively. Finally, the differential level and trend before and after the FDA warning revision between $C_{ij} = 1$ and $C_{ij} = 0$ are σ and δ, respectively.

eTable 1. Association of FDA Warnings With Fluoroquinolone Prescriptions Across Three Acute Indications

	Model: sinusitis patients		Model: bronchitis patients		Model: uUTI patients	
	[1]	[2]	[3]	[4]	[5]	[6]
	Coefficient/CI	*p*-value	Coefficient/CI	*p*-value	Coefficient/CI	*p*-value
Baseline Trend	-0.2277	(<0.001)	-0.2164	(<0.001)	0.0069	(0.350)
	[-0.2337,-0.2217]		[-0.2261,-0.2067]		[-0.0075,0.0213]	
Change in Trend	0.198	(<0.001)	0.1717	(<0.001)	-0.0385	(<0.001)
(Post period 1 Vs. Baseline)	[0.1901,0.2059]		[0.1608,0.1827]		[-0.0595,-0.0174]	
Change in Trend	0.0589	(<0.001)	0.0677	(<0.001)	-0.0419	(0.071)
(Post period 2 Vs. Post period 1)	[0.0439,0.0738]		[0.0580,0.0774]		[-0.0874,0.0036]	
Change in Level	1.94	(<0.001)	1.9564	(<0.001)	2.8438	(<0.001)
(Post period 1 Vs. Baseline)	[1.8050,2.0751]		[1.7561,2.1567]		[2.4426,3.2450]	
Change in Level	-0.3293	(<0.001)	-0.102	(0.061)	-2.9217	(<0.001)
(Post period 2 Vs. Post period 1)	[-0.5124,-0.1462]		[-0.2085,0.0045]		[-3.4829,-2.3605]	

Notes: All regressions are controlled for patient age, sex, indicators for race, elixhauser index, indicators for the four U.S. Census regions (i.e., the Northeast, the Midwest, the South, and the West), month fixed effects, and the five physician affiliations. The standard errors are clustered at the patient level. The models show results from regressions that exclude patients with complicated UTI. Columns [1] and [2] represent the model for only patients with sinusitis indications, [3] and [4] with bronchitis indications, and [5] and [6] with uncomplicated UTI condition.

© 2021 Sankar A et al. *JAMA Network Open.*

eTable 2. Differential Association of Physician Affiliations After FDA Warnings With Fluoroquinolone Prescriptions for Patients With Sinusitis

Model 1: Difference for PCP relative to non-PCP physicians		
	Coefficient/CI	*p*-value
Baseline Trend	-0.0349	(<0.001)
	[-0.0497,-0.0200]	
Change in Trend	0.0318	(0.001)
(Post period 1 Vs. Baseline)	[0.0126,0.0509]	
Change in Trend	-0.0142	(0.433)
(Post period 2 Vs. Post period 1)	[-0.0498,0.0214]	
Change in Level	0.3874	(0.024)
(Post period 1 Vs. Baseline)	[0.0510,0.7237]	
Change in Level	-0.0086	(0.969)
(Post period 2 Vs. Post period 1)	[-0.4477,0.4304]	
Model 2: Difference for IDN relative to non-IDN physicians		
Baseline Trend	-0.0031	(0.730)
	[-0.0209,0.0147]	
Change in Trend	0.0039	(0.777)
(Post period 1 Vs. Baseline)	[-0.0228,0.0305]	
Change in Trend	-0.0315	(0.396)
(Post period 2 Vs. Post period 1)	[-0.1044,0.0413]	
Change in Level	0.0688	(0.754)
(Post period 1 Vs. Baseline)	[-0.3616,0.4992]	
Change in Level	0.4575	(0.311)
(Post period 2 Vs. Post period 1)	[-0.4271,1.3421]	
Model 3: Difference for teaching relative to		
non-teaching physicians		
Baseline Trend	0.0273	(<0.001)
	[0.0153,0.0392]	
Change in Trend	-0.0229	(0.004)
(Post period 1 Vs. Baseline)	[-0.0385,-0.0073]	

© 2021 Sankar A et al. *JAMA Network Open.*

Change in Trend	0.0071	(0.635)
(Post period 2 Vs. Post period 1)	[-0.0222,0.0365]	
Change in Level	-0.5292	(<0.001)
(Post period 1 Vs. Baseline)	[-0.7977,-0.2606]	
Change in Level	-0.1116	(0.543)
(Post period 2 Vs. Post period 1)	[-0.4711,0.2479]	
Model 4: Difference for for-profit relative to		
non-for-profit hospital physicians		
Baseline Trend	-0.0342	(<0.001)
	[-0.0466,-0.0219]	
Change in Trend	0.0271	(0.001)
(Post period 1 Vs. Baseline)	[0.0110,0.0432]	
Change in Trend	-0.0167	(0.277)
(Post period 2 Vs. Post period 1)	[-0.0468,0.0134]	
Change in Level	0.4942	(0.001)
(Post period 1 Vs. Baseline)	[0.2152,0.7732]	
Change in Level	0.2789	(0.138)
(Post period 2 Vs. Post period 1)	[-0.0895,0.6473]	
Model 5: Differences for the physicians affiliated to the		
top 10th percentile CMI level as compared to those not in top 10th percentile		
Baseline Trend	0.0366	(0.001)
	[0.0154,0.0577]	
Change in Trend	-0.0186	(0.164)
(Post period 1 Vs. Baseline)	[-0.0448,0.0076]	
Change in Trend	0.0149	(0.392)
(Post period 2 Vs. Post period 1)	[-0.0192,0.0491]	
Change in Level	-0.5053	(0.032)
(Post period 1 Vs. Baseline)	[-0.9669,-0.0436]	
Change in Level	-0.8257	(<0.001)
(Post period 2 Vs. Post period 1)	[-1.2676,-0.3838]	

Notes: All regressions are controlled for patient age, sex, indicators for race, elixhauser index, indicators for the four U.S. Census regions (i.e., the Northeast, the Midwest, the South, and the West), month fixed effects, and the five

© 2021 Sankar A et al. *JAMA Network Open.*

physician affiliations. The standard errors are clustered at the patient level. The models show results from regressions that exclude patients with complicated UTI.

© 2021 Sankar A et al. *JAMA Network Open.*

eTable 3. Differential Association of Physician Affiliations After FDA Warnings With Fluoroquinolone Prescriptions for Patients With Bronchitis

Model 1: Difference for PCP relative to non-PCP physicians		
	Coefficient/CI	*p*-value
Baseline Trend	-0.0936	(<0.001)
	[-0.1129,-0.0743]	
Change in Trend	0.1022	(<0.001)
(Post period 1 Vs. Baseline)	[0.0805,0.1238]	
Change in Trend	-0.0079	(0.359)
(Post period 2 Vs. Post period 1)	[-0.0248,0.0090]	
Change in Level	0.5005	(0.014)
(Post period 1 Vs. Baseline)	[0.1031,0.8979]	
Change in Level	-0.2863	(0.005)
(Post period 2 Vs. Post period 1)	[-0.4853,-0.0874]	
Model 2: Difference for IDN relative to non-IDN physicians		
Baseline Trend	0.0072	(0.653)
	[-0.0241,0.0384]	
Change in Trend	-0.0079	(0.669)
(Post period 1 Vs. Baseline)	[-0.0443,0.0284]	
Change in Trend	0.0139	(0.541)
(Post period 2 Vs. Post period 1)	[-0.0308,0.0587]	
Change in Level	0.3981	(0.239)
(Post period 1 Vs. Baseline)	[-0.2652,1.0614]	
Change in Level	-0.1793	(0.523)
(Post period 2 Vs. Post period 1)	[-0.7290,0.3704]	
Model 3: Difference for teaching relative to		
non-teaching physicians		
Baseline Trend	0.0469	(<0.001)
	[0.0274,0.0663]	
Change in Trend	-0.0505	(<0.001)
(Post period 1 Vs. Baseline)	[-0.0723,-0.0287]	
Change in Trend	-0.0013	(0.887)

© 2021 Sankar A et al. *JAMA Network Open.*

(Post period 2 Vs. Post period 1)	[-0.0185,0.0160]	
Change in Level	-0.3191	(0.115)
(Post period 1 Vs. Baseline)	[-0.7156,0.0773]	
Change in Level	0.1763	(0.090)
(Post period 2 Vs. Post period 1)	[-0.0273,0.3799]	
Model 4: Difference for for-profit relative to		
non-for-profit hospital physicians		
Baseline Trend	-0.0166	(0.105)
	[-0.0367,0.0035]	
Change in Trend	0.0232	(0.043)
(Post period 1 Vs. Baseline)	[0.0007,0.0458]	
Change in Trend	-0.011	(0.225)
(Post period 2 Vs. Post period 1)	[-0.0287,0.0067]	
Change in Level	0.1033	(0.623)
(Post period 1 Vs. Baseline)	[-0.3084,0.5150]	
Change in Level	-0.1204	(0.260)
(Post period 2 Vs. Post period 1)	[-0.3299,0.0892]	
Model 5: Differences for the physicians affiliated to the		
top 10th percentile CMI level as compared to those not in top 10th percentile		
Baseline Trend	-0.0041	(0.860)
	[-0.0499,0.0417]	
Change in Trend	0.0236	(0.347)
(Post period 1 Vs. Baseline)	[-0.0255,0.0727]	
Change in Trend	-0.0241	(0.059)
(Post period 2 Vs. Post period 1)	[-0.0492,0.0010]	
Change in Level	-0.4065	(0.348)
(Post period 1 Vs. Baseline)	[-1.2553,0.4422]	
Change in Level	0.1068	(0.472)
(Post period 2 Vs. Post period 1)	[-0.1838,0.3973]	

Notes: All regressions are controlled for patient age, sex, indicators for race, elixhauser index, indicators for the four U.S. Census regions (i.e., the Northeast, the Midwest, the South, and the West), month fixed effects, and the five

© 2021 Sankar A et al. *JAMA Network Open.*

physician affiliations. The standard errors are clustered at the patient level. The models show results from regressions that exclude patients with complicated UTI.

© 2021 Sankar A et al. *JAMA Network Open.*

eTable 4. Differential Association of Physician Affiliations After FDA Warnings With Fluoroquinolone Prescriptions for Patients With Uncomplicated UTI

Model 1: Difference for PCP relative to non-PCP physicians		
	Coefficient/CI	*p*-value
Baseline Trend	0.0406	(0.004)
	[0.0128,0.0683]	
Change in Trend	0.0441	(0.033)
(Post period 1 Vs. Baseline)	[0.0036,0.0846]	
Change in Trend	-0.1673	(<0.001)
(Post period 2 Vs. Post period 1)	[-0.2532,-0.0815]	
Change in Level	0.9932	(0.012)
(Post period 1 Vs. Baseline)	[0.2196,1.7668]	
Change in Level	-0.016334	(0.003)
(Post period 2 Vs. Post period 1)	[-0.026952,-0.5716]	
Model 2: Difference for IDN relative to non-IDN physicians		
Baseline Trend	0.012	(0.610)
	[-0.0342,0.0582]	
Change in Trend	0.0507	(0.207)
(Post period 1 Vs. Baseline)	[-0.0281,0.1294]	
Change in Trend	-0.1234	(0.289)
(Post period 2 Vs. Post period 1)	[-0.3516,0.1048]	
Change in Level	-0.4099	(0.550)
(Post period 1 Vs. Baseline)	[-0.017531,0.9332]	
Change in Level	2.15	(0.159)
(Post period 2 Vs. Post period 1)	[-0.7826,4.7856]	
Model 3: Difference for teaching relative to		
non-teaching physicians		
Baseline Trend	0.0509	(<0.001)
	[0.0223,0.0795]	
Change in Trend	-0.0779	(<0.001)
(Post period 1 Vs. Baseline)	[-0.1199,-0.0358]	

© 2021 Sankar A et al. *JAMA Network Open.*

Change in Trend	-0.001	(0.982)
(Post period 2 Vs. Post period 1)	[-0.0908,0.0888]	
Change in Level	-0.3158	(0.437)
(Post period 1 Vs. Baseline)	[-1.1129,0.4812]	
Change in Level	0.7795	(0.167)
(Post period 2 Vs. Post period 1)	[-0.3261,1.8852]	
Model 4: Difference for for-profit relative to		
non-for-profit hospital physicians		
Baseline Trend	-0.0039	(0.794)
	[-0.0331,0.0253]	
Change in Trend	-0.025	(0.252)
(Post period 1 Vs. Baseline)	[-0.0678,0.0178]	
Change in Trend	0.0494	(0.281)
(Post period 2 Vs. Post period 1)	[-0.0405,0.1394]	
Change in Level	0.1863	(0.655)
(Post period 1 Vs. Baseline)	[-0.6307,1.32]	
Change in Level	0.9989	(0.078)
(Post period 2 Vs. Post period 1)	[-0.11242.1102]	
Model 5: Differences for the physicians affiliated to the		
top 10th percentile CMI level as compared to those not in top 10th percentile		
Baseline Trend	-0.0108	(0.705)
	[-0.0664,0.0449]	
Change in Trend	0.0313	(0.410)
(Post period 1 Vs. Baseline)	[-0.0431,0.1057]	
Change in Trend	-0.0198	(0.713)
(Post period 2 Vs. Post period 1)	[-0.1256,0.0859]	
Change in Level	-1.2405	(0.095)
(Post period 1 Vs. Baseline)	[-2.6989,0.2179]	
Change in Level	-0.1187	(0.866)
(Post period 2 Vs. Post period 1)	[-1.4972,1.2597]	

© 2021 Sankar A et al. *JAMA Network Open.*

Notes: All regressions are controlled for patient age, sex, indicators for race, elixhauser index, indicators for the four U.S. Census regions (i.e., the Northeast, the Midwest, the South, and the West), month fixed effects, and the five physician affiliations. The standard errors are clustered at the patient level. The models show results from regressions that exclude patients with complicated UTI.

© 2021 Sankar A et al. *JAMA Network Open.*

eTable 5. Differential Association of PCPs Across Different Institutional Affiliations

	Coefficient/CI	*p*-value
Interaction of baseline trend with:		
PCP	-0.0972	(<0.001)
	[-0.1143,-0.0802]	
PCP X top 10th percentile CMI	0.0118	(0.171)
	[-0.0051,0.0287]	
PCP X IDN	0.0127	(0.068)
	[-0.0010,0.0263]	
PCP X teaching	0.0176	(<0.001)
	[0.0092,0.0260]	
PCP X for-profit	-0.0048	(0.269)
	[-0.0132,0.0037]	
Interaction of post period 1 trend with:		
PCP	0.1316	(<0.001)
	[0.1030,0.1602]	
PCP X top 10th percentile CMI	-0.0098	(0.506)
	[-0.0387,0.0191]	
PCP X IDN	0.0004	(0.978)
	[-0.0260,0.0267]	
PCP X teaching	-0.0389	(<0.001)
	[-0.0543,-0.0236]	
PCP X for-profit	-0.0114	(0.141)
	[-0.0266,0.0038]	
Interaction of post period 2 trend with:		
PCP	0.0748	(0.034)
	[0.0055,0.1440]	
PCP X top 10th percentile CMI	-0.0209	(0.341)
	[-0.0640,0.0222]	
PCP X IDN	-0.0295	(0.399)
	[-0.0981,0.0390]	

© 2021 Sankar A et al. *JAMA Network Open.*

PCP X teaching	0.0151	(0.358)
	[-0.0171,0.0474]	
PCP X for-profit	0.0023	(0.883)
	[-0.0287,0.0333]	
Interaction of post period 1 level with:		
PCP	0.5808	(0.040)
	[0.0260,1.1355]	
PCP X top 10th percentile CMI	-0.528	(0.143)
	[-1.2344,0.1783]	
PCP X IDN	0.1843	(0.481)
	[-0.3284,0.6970]	
PCP X teaching	0.1128	(0.510)
	[-0.2225,0.4480]	
PCP X for-profit	0.1054	(0.536)
	[-0.2286,0.4393]	
Interaction of post period 2 level with:		
PCP	-1.6945	(<0.001)
	[-2.5293,-0.8596]	
PCP X top 10th percentile CMI	0.5288	(0.056)
	[-0.0131,0.010708]	
PCP X IDN	0.2172	(0.606)
	[-0.6084,1.0428]	
PCP X teaching	0.2619	(0.187)
	[-0.1268,0.6505]	
PCP X for-profit	0.451	(0.019)
	[0.0753,0.8266]	

Notes: The regression is controlled for patient age, sex, indicators for race, elixhauser index, indicators for the four U.S. Census regions (i.e., the Northeast, the Midwest, the South, and the West), month fixed effects, and the five physician affiliations. The standard errors are clustered at the patient level. The model show results from regressions that exclude patients with complicated UTI.

© 2021 Sankar A et al. *JAMA Network Open.*

eTable 6. Sensitivity Analysis With Different Washout Periods

		Baseline Trend	Change in Trend	Change in Trend	Change in Level	Change in Level
			(Post period 1	(Post period 2	(Post period 1	(Post period 2
Models			Versus Baseline)	Versus Baseline)	Versus Baseline)	Versus Baseline)
7-month	Coefficient	-0.183	0.085	0.062	3.426	-0.770
washout period	*p-value*	(<0.001)	(<0.001)	(<0.001)	(<0.001)	(<0.001)
**(Main design)**	CI	[-0.190,-0.176]	[0.075,0.095]	[0.0428,0.0807]	[3.23,3.623]	[-0.995,-0.544]
9-month	Coefficient	-0.196	0.101	0.081	3.716	-1.167
washout period	*p-value*	(<0.001)	(<0.001)	(<0.001)	(<0.001)	(<0.001)
	CI	[-0.2033, -0.1890]	[0.0906, 0.1122]	[0.0602,0.1025]	[3.4963,3.9347]	[-1.4319,-0.9012]
5-month	Coefficient	-0.173	0.072	0.059	3.200	-0.640
washout period	*p-value*	(<0.001)	(<0.001)	(<0.001)	(<0.001)	(<0.001)
	CI	[-0.1793,-0.1662]	[0.0630,0.0815]	[0.0413,0.0760]	[3.0176,3.3820]	[-0.8412,-0.4395]
3 month	Coefficient	-0.165	0.058	0.070	3.112	-0.578
washout period	*p-value*	(<0.001)	(<0.001)	(<0.001)	(<0.001)	(<0.001)
	CI	[-0.1713,-0.1586]	[0.0495,0.0671]	[0.0539,0.0860]	[2.9398,3.2844]	[-0.7623,-0.3931]

Notes: All regressions are controlled for patient age, sex, indicators for race, elixhauser index, indicators for the four U.S. Census regions (i.e., the Northeast, the Midwest, the South, and the West), month fixed effects, and the five physician affiliations. The standard errors are clustered at the patient level. The models show results from regressions that exclude patients with complicated UTI. Each row represents a separate regression model for different washout periods.

© 2021 Sankar A et al. *JAMA Network Open.*
